# Monitoring deformation of seasonally frozen Yellow River bank soil: A synergetic application of the SBAS InSAR approach

**DOI:** 10.1371/journal.pone.0339366

**Published:** 2025-12-26

**Authors:** Yuxin Mao, Honglan Ji, Zhen Yang

**Affiliations:** 1 College of Water Resources and Civil Engineering, Inner Mongolia Agricultural University, Hohhot, Inner Mongolia, China; 2 Collaborative Innovation Center for Comprehensive Management of Water Resources and Water Environment in Inner Mongolia of the Yellow River Basin, Hohhot, Inner Mongolia, China; 3 State Key Laboratory of Water Engineering Ecology and Environment in Arid Area, Inner Mongolia Agricultural University, Hohhot, Inner Mongolia, China; Indian Institute of Technology Jammu, INDIA

## Abstract

The riverbanks of the Inner Mongolia reach of the Yellow River face persistent risks of deformation and collapse due to freeze-thaw cycles and hydrodynamic forces. However, most existing studies have concentrated on urban land subsidence or the stability of hydraulic structures, while long-term and multi-reach systematic monitoring of this seasonally frozen and meandering section remains scarce. Based on Sentinel-1A satellite data, this study employs the Small Baseline Subset InSAR (SBAS-InSAR) technique to derive the spatiotemporal characteristics of riverbank deformation, integrates channel migration information to examine the spatial relationship between channel evolution and bank deformation, and conducts long-term deformation pattern analyses in representative reaches. The results indicate that the riverbanks in this section are generally stable, with 96.5% of monitoring points showing an average annual deformation rate within ±30 mm/a. Nevertheless, significant deformation was detected in some areas between the Bayangaole and Toudaoguai hydrological stations, dominated by uplift and strongly influenced by river water level fluctuations. Subsidence primarily occurred along the outer banks of meander bends, where loose bank structures pose potential stability risks. Specifically, in the Shisifenzi area, the average annual deformation rate ranged from −20.1 to 24.8 mm/a, with subsidence concentrated at meander crests and cultivated land, potentially affecting levee stability and farmland safety. In the Wenbuhao area, rates ranged from −18.7 to 19.5 mm/a, with subsidence concentrated along the eastern riverbank, indicating localized erosion risks, while uplift mainly occurred in farmland farther from the river. This study reveals the differentiated response characteristics of various reaches under hydrodynamic forces and demonstrates that SBAS-InSAR is effective for monitoring riverbank deformation in complex environments. The findings provide reliable technical support for riverbank hazard prevention and the protection of riverfront infrastructure in seasonally frozen regions.

## 1. Introduction

The Inner Mongolia reach of the Yellow River is a seasonally frozen river system in northern China. Its riverbanks are subject not only to prolonged fluvial erosion but also to freeze-thaw cycles in winter, which degrade the soil’s physical and mechanical properties, leading to gradual subsidence and eventual structural failure [1]. These collapses compromise embankment stability, agricultural land, and transportation infrastructure, while also exacerbating sedimentation and soil loss, posing a significant threat to riverside communities [2]. Therefore, high-precision deformation monitoring is essential for slope stability assessment and collapse prediction.

In recent years, disaster prevention and mitigation have garnered widespread attention. Accurate and scientific monitoring of riverbank deformation and early disaster warning have become critical challenges in water conservancy management and disaster prevention. However, in river basins spanning hundreds to thousands of kilometers, traditional field survey methods are constrained by high costs and prolonged on-site operations, making it difficult to meet large-scale monitoring demands with high spatiotemporal resolution [3,4]. The rapid advancement of Interferometric Synthetic Aperture Radar (InSAR) technology has introduced a novel solution for riverbank deformation monitoring [5]. InSAR technology offers all-weather, continuous observation capabilities, enabling large-scale monitoring and making it particularly suitable for regions where traditional ground-based monitoring equipment is difficult to deploy. Consequently, it has become an effective supplementary approach for surface deformation monitoring [6].

Differential InSAR (D-InSAR) [7] technology has rapidly developed based on conventional InSAR. By performing interferometric analysis on two SAR images, it can achieve millimeter-level accuracy in surface deformation detection. However, it is easily affected by temporal and spatial decorrelation and atmospheric phase delays, making it difficult to achieve stable long-term monitoring of subtle deformations. To overcome the issue of decorrelation, Persistent Scatterer InSAR (PS-InSAR) [8] and Small Baseline Subset InSAR (SBAS-InSAR) [9] techniques were subsequently proposed to enhance the capability of long-term deformation monitoring. The PS-InSAR technique achieves high-precision deformation monitoring by identifying temporally stable scatterers (PS points), showing significant advantages in highly coherent areas such as urban environments or bare ground surfaces, with monitoring accuracy reaching the millimeter level. However, in regions characterized by dense vegetation, extensive water bodies, or frequent surface changes, the interferometric phase is easily affected by complex scattering mechanisms, making it difficult to identify a sufficient number of stable scatterers. This limits the effectiveness and reliability of PS-InSAR results. In contrast, the SBAS-InSAR technique constructs multiple small-baseline interferometric pairs and conducts time-series analysis, allowing for the identification of pixels that maintain long-term coherence in complex surface environments. This enables the extraction of more stable ground deformation signals. Its multi-temporal characteristics contribute to noise suppression and accuracy improvement, making it more suitable and reliable than conventional D-InSAR in areas with significant topographic variation and heterogeneous vegetation coverage. This approach has been extensively applied to the monitoring of landslide hazards [10,11], mining subsidence [12,13], and urban ground subsidence [14,15]. Furthermore, numerous studies have demonstrated the feasibility of using InSAR technology for high-precision deformation monitoring of hydraulic structures and in complex geomorphological environments. For instance, Zhou W et al. [16] conducted deformation monitoring of the Shuibuya Dam using ALOS PALSAR data, and the results showed a high correlation of 0.92 between the InSAR-derived measurements and leveling data, confirming the feasibility of applying InSAR technology in dam stability monitoring. Wosowski et al. [17] combined InSAR techniques with COSMO-SkyMed imagery to monitor the Disueri earth dam and the Gela breakwater. Their results indicated that the breakwater experienced a deformation rate of up to 40 mm/year during the period from 2011 to 2016, demonstrating the potential of InSAR for long-term monitoring of dam and embankment deformation. In the same year, Emadali et al. [18] analyzed the deformation of the Masjed-Soleyman Dam based on TerraSAR-X data. The results were consistent with field observations in terms of subsidence trends, further validating the applicability of InSAR for monitoring dam deformation. Awasthi et al. [19,20] employed the PS-InSAR technique to investigate surface deformation in the Himalayan foothill and the city of Lucknow, India, systematically revealing the coupled relationships between surface deformation and factors such as land use changes, excessive groundwater extraction, intense precipitation, and topographic undulations. Their study highlighted the potential of InSAR technology for geological hazard identification and early warning. Varade et al. [21] explored the application of differential interferometric phase and Sentinel-1 dual-polarization data in snow depth estimation, demonstrating the adaptability and reliability of this technique in complex surface environments of plateau regions.

Although InSAR technology has been widely applied to deformation monitoring of revetment projects, dams, and various complex geomorphic areas, the riverbanks of the Inner Mongolia section of the Yellow River are subjected to multiple interacting factors, including freeze-thaw cycles, hydrodynamic erosion, and groundwater fluctuations, leading to diverse and complex deformation mechanisms of both revetment structures and soils. Therefore, conducting InSAR-based deformation monitoring is of great significance for revealing the spatiotemporal characteristics of riverbank deformation and assessing potential risks. In this study, the SBAS-InSAR technique was applied to Sentinel-1 radar imagery to monitor riverbank areas within 5 km on both sides of the Yellow River in the Inner Mongolia section, obtaining the spatiotemporal distribution characteristics of surface deformation from 2021 to 2022. By integrating river channel migration information extracted from Sentinel-2 optical imagery, representative river sections were identified for long-term deformation monitoring. Additionally, a comprehensive deformation analysis was conducted for the Shisifenzi and Wenbuhao areas, covering the period from 2018 to 2023. The monitoring results were evaluated and validated against leveling measurement data to assess the accuracy and feasibility of SBAS-InSAR technology for riverbank deformation monitoring. This study presents a reliable technical approach for safety monitoring and hazard identification along the Yellow River, offering valuable insights into riverbank stability assessment and disaster prevention.

## 2. Study area and data description

### 2.1. Study area overview

**Fig 1:**
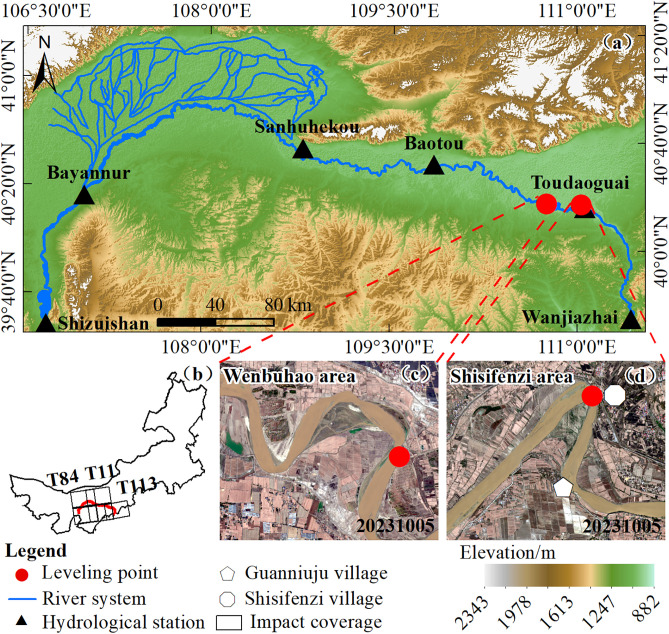
Overview of the study area. **(a)** Distribution of hydrological stations along the Inner Mongolia section of the Yellow River. **(b)** Coverage of Sentinel-1 SAR imagery. **(c)** Location of the Shisifenzi area. **(d)** Location of the Wenbuhao area. The background imagery in panels **(c)** and **(d)** is based on Sentinel-2 data provided by the Copernicus Data Space Ecosystem (CDSE, https://dataspace.copernicus.eu).

**Fig 2:**
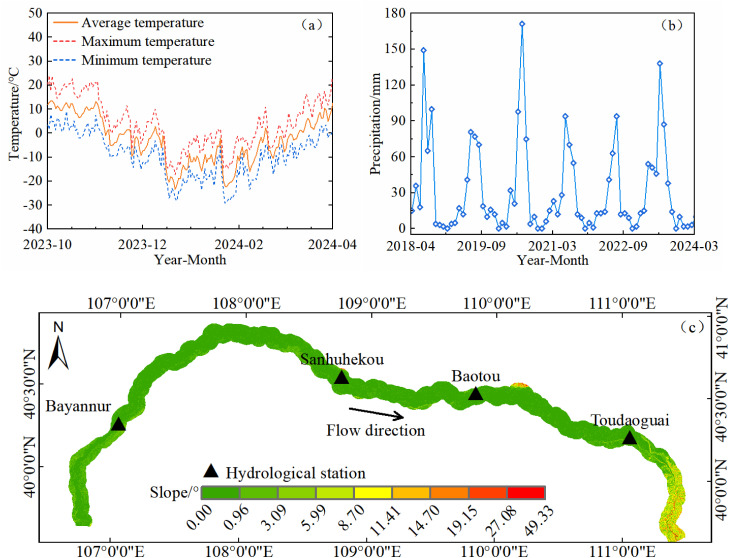
Climate and topographic conditions of the study area. **(a)** Temperature, **(b)** Precipitation, **(c)** Slope.

The Inner Mongolia section of the Yellow River is located at the northernmost part of the Yellow River Basin, spanning from 37°30′ to 41°50′ N and 106°00′ to 113°00′ E. The main river channel runs approximately 830 km in length, entering the Inner Mongolia Autonomous Region from Shizuishan in Ningxia and exiting at Hekou Town, south of the Wanjiazhai Reservoir [22]. This river section flows through major geomorphological units such as the Loess Plateau, the Ordos Plateau, and the Hetao Plain. Along the river, five major hydrological stations are distributed: Shizuishan, Bayangaole, Sanhuhekou, Baotou, and Toudaoguai. Influenced by topography and channel morphology, the river section between Shizuishan and Baotou exhibits pronounced channel migration and alternating erosion and deposition along the banks [23]. This study selects two typical meander bends as monitoring areas: the Shisifenzi bend (40°17′39″ N, 111°2′53″ E) and the Wenbuhao bend (40°17′30″ N, 111°46′15″ E), both located between the Baotou and Toudaoguai hydrological stations (Fig 1). This segment of the river is predominantly sinuous, and both riverbanks are reinforced with gabion revetments, which effectively restrict lateral channel migration and provide stable observation conditions for long-term InSAR deformation monitoring. The region experiences an average annual precipitation of 150–460 mm, with significant interannual variability and uneven spatial and temporal distribution. Approximately 70% of the annual rainfall occurs between July and September, with a decreasing gradient from southeast to northwest. The annual evaporation ranges from 1,200–2,000 mm, far exceeding the precipitation, indicating an arid to semi-arid climate characterized by low rainfall, intense evaporation, and prolonged sunshine duration [1,24,25]. Seasonal changes are also distinct: temperatures typically fall below freezing by mid-November and rise above freezing by mid-March, resulting in a freeze-thaw period of about 130 days, classifying the region as a typical seasonally frozen soil area. The overall riverbank topography is relatively flat, with slopes mainly ranging from 0° to 4° (Fig 2). The surface relief is minimal, which is favorable for maintaining interferometric coherence, and also indicates that riverbank deformation is dominated by the vertical component, with relatively minor influence from horizontal displacement. The riverbank soil mainly consists of silt and sand, primarily composed of alluvium transported from the upstream Loess Plateau of the Yellow River. These are Quaternary unconsolidated sediments with strong collapsibility [26]. In addition, the study area is a typical agricultural zone, where spring irrigation heavily relies on Yellow River diversion and groundwater recharge. Due to the uneven spatial and temporal distribution of water resources, groundwater levels fluctuate frequently during both the irrigation and freezing periods, significantly affecting the stability of the riverbank soil.

### 2.2. Data sources

A total of 386 Sentinel-1 Single Look Complex (SLC) radar images were used in this study, covering track numbers 11, 84, and 113. These tracks encompass both sides of the Yellow River channel within a 5 km buffer zone along the Inner Mongolia section. Due to the data availability of the Sentinel-1A satellite, only ascending orbit images can be obtained for the study area. Although a single orbit direction cannot fully reconstruct three-dimensional deformation information, the relatively gentle topography and the predominance of vertical subsidence and uplift along the riverbanks enable ascending images to effectively capture the primary deformation patterns. Therefore, SAR imagery acquired between January 2021 and December 2022 was used to monitor deformation within the study area and to identify zones of significant surface displacement. On this basis, a longer time span of imagery from April 2018 to April 2024 was further employed to conduct time-series inversion in these areas, aiming to reveal the long-term deformation evolution.

Topographic phase was removed using the Shuttle Radar Topography Mission Digital Elevation Model (SRTM-DEM) with a spatial resolution of 30 m. Tropospheric delay effects were corrected using delay products provided by the Generic Atmospheric Correction Online Service for InSAR (GACOS). The monthly mean precipitation data used in this study were obtained from the National Earth System Science Data Center (https://www.geodata.cn). This dataset is derived from global climate data and high-resolution climate records through spatial downscaling, with a spatial resolution of approximately 1 km, which can effectively represent the regional precipitation distribution characteristics of the study area. River water level data were obtained from the Yellow River Conservancy Commission (http://www.yrcc.gov.cn). Leveling and groundwater data were collected using settlement sensors and groundwater level meters deployed near the Shisifenzi and Wenbuhao riverbanks. In addition, multispectral Sentinel-2 optical imagery was used to assist with land cover identification and to extract changes in riverbank boundaries. These data were obtained from the Copernicus Open Access Hub (https://scihub.copernicus.eu), and the acquisition dates of the images are annotated in the figures for reference to different stages of river channel morphology. All remote sensing and auxiliary data used in this study strictly comply with the usage terms and policies of their respective providers, including the European Space Agency (ESA) Copernicus Open Access Policy and the data usage agreements of national data service platforms.

## 3. Research methods

**Fig 3:**
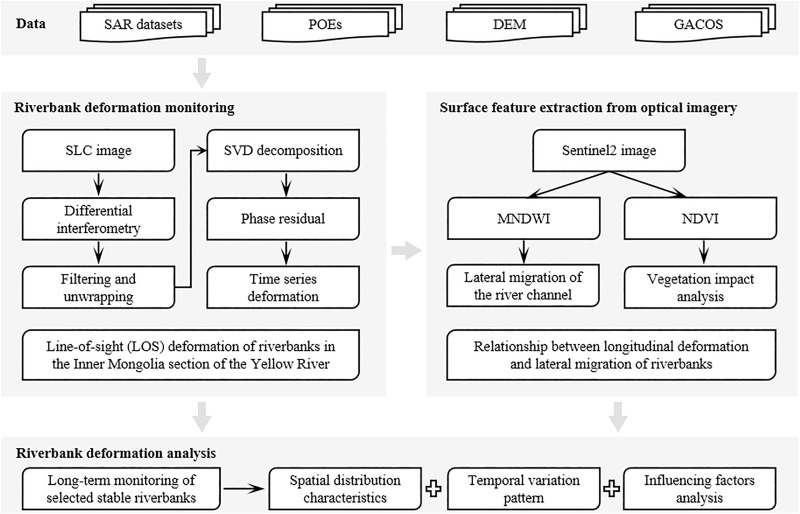
Workflow for riverbank deformation monitoring and analysis.

This study is based on ascending-pass Sentinel-1 SAR images and optical remote sensing data, and establishes a comprehensive technical framework for riverbank deformation monitoring and analysis in the Inner Mongolia section of the Yellow River. First, LOS (line-of-sight) surface deformation data of the riverbanks in the study area are obtained using SBAS-InSAR technology. Second, river channel boundaries are extracted from Sentinel-2 optical images using the MNDWI water index, and the spatial correspondence between river morphology and riverbank deformation is analyzed. Finally, typical river sections such as Shisifenzi and Wenbuhao are selected to analyze the spatiotemporal distribution characteristics of riverbank time-series deformation, and the influencing mechanisms are discussed in combination with precipitation, river water level, and groundwater level variations. The overall technical workflow of the study is shown in Fig 3.

### 3.1. Deformation monitoring of riverbanks based on time-series InSAR technology

SBAS-InSAR is a time-series interferometric SAR technique based on multiple master images. It reduces the effects of spatiotemporal decorrelation by selecting interferometric pairs with small spatial and temporal baselines, and it retrieves continuous deformation time series through least-squares inversion. Assuming that *N* + 1 SAR images covering the same area are acquired at times (*t*_0_,*t*_1_,…,*t*_*N*_), radar image pairs are constructed based on spatiotemporal baseline thresholds to generate *M* differential interferograms, where *M* satisfies:


$$\frac{N+1}{\text{2}}\leq M\leq \frac{N(N+1)}{\text{2}}$$ (1)

For the *j*-th unwrapped differential interferogram, the unwrapped phase of pixel (*x*,*r*) can be expressed as:


$$\delta \phi_{j}(x,r)=\phi (t_{B},x,r)-\phi (t_{A},x,r)\approx \delta \phi_{j}^{topo}(x,r)+\delta \phi_{j}^{disp}(x,r)+\delta \phi_{j}^{atm}(x,r)+\delta \phi_{j}^{noise}(x,r)$$ (2)

where ϕ is the interferometric phase; *t_A_* and *t_B_* are the acquisition times of the master and slave images of the *j*-th interferogram, respectively; $\delta \phi_{j}^{topo}(x,r)$ is the phase caused by DEM errors; $\delta \phi_{j}^{disp}(x,r)$ is the phase induced by surface deformation; $\delta \phi_{j}^{aim}(x,r)$ is the atmospheric phase delay at *t_A_* and *t_B_*; and $\delta \phi_{j}^{noise}(x,r)$ is the phase caused by noise.

From the obtained interferograms, equations are formulated to link the deformation variables at different acquisition times with the corresponding differential interferometric phases, yielding *M* linear equations that can be expressed in matrix form as:


δϕ(x,r)=Aϕ(x,r) (3)

where *A* is the *M* × *N* coefficient matrix, and ϕ(x,r) represents the unknown deformation phases at *N* time points for pixel (*x*,*r*). When *M* ≥ *N*, the least-squares solution can be obtained as:


$$\phi (x,r)=(A^{T}A{)}^{-1}A^{T}\delta \phi (x,r)$$ (4)

When M < N, the system has infinitely many solutions. Singular Value Decomposition (SVD) is used to jointly solve multiple small baselines, ultimately obtaining the cumulative deformation for each time step.

Based on the SBAS-InSAR principle described above, this study utilizes ascending-pass Sentinel-1 SAR data in combination with the open-source platforms Sentinel Application Platform (SNAP) and the StaMPS toolbox to monitor riverbank deformation in the Inner Mongolia section of the Yellow River. The data used are VV-polarized products, which, compared to VH polarization, offer higher backscattering intensity and interferometric coherence. VV polarization performs more stably in areas with relatively flat terrain, sparse vegetation cover, or near riverbanks and water bodies, making it more suitable for surface deformation monitoring. Interferogram preprocessing is conducted in SNAP, including orbit correction, image co-registration, interferogram generation, and removal of topographic phase using the SRTM DEM. To improve the signal-to-noise ratio, Goldstein filtering and 4 × 1 multilooking were applied. The temporal baseline of interferometric pairs was constrained within 120 days, while the spatial baseline was limited to 2% of the critical baseline (approximately 150 m), less than 3 m, in order to balance high coherence with sensitivity to seasonal deformation. A total of 499 interferograms were generated, covering Tracks 11, 84, and 113. The interferogram network and its temporal-spatial baseline distribution are shown in Fig 3, illustrating the connections among different SAR acquisitions in both time and space.

**Fig 4:**
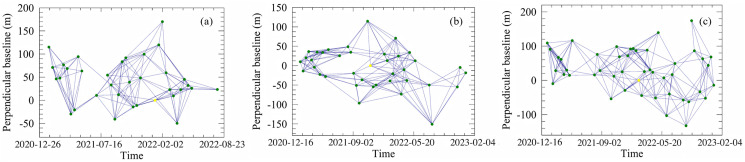
Spatiotemporal baseline distribution of S1 SAR data for relative orbits. **(a)** Track 11. **(b)** Track 84. **(c)** Track 113.

During coherence screening, a threshold of 0.4 was adopted to ensure sufficient coverage of monitoring points and the spatial continuity of the time-series results. This threshold effectively removes low-quality interferometric points while retaining an adequate number of pixels, thereby reducing uncertainties introduced by noise and unwrapping errors. Since interferometric phases are wrapped within the range of [−π, π], phase unwrapping was required to reconstruct a continuous phase field. Accordingly, the Minimum Cost Flow (MCF) algorithm in SNAP was employed. The MCF method performs more stably in low-coherence regions (e.g., areas with vegetation cover or near rivers) and reduces phase jump errors compared with least-squares unwrapping. Combined with coherence thresholding, this approach enhances unwrapping accuracy and success rates under complex surface conditions. The unwrapped results were imported into StaMPS for time-series inversion, and tropospheric delay corrections were performed using GACOS atmospheric products, effectively suppressing long-wavelength atmospheric artifacts Fig 4. Finally, the least-squares method combined with Singular Value Decomposition (SVD) is applied to extract the deformation time series and the average line-of-sight (LOS) deformation velocity, enabling stable and accurate deformation monitoring [27–29].

### 3.2. Surface feature recognition based on optical imagery

The riverbanks of the Inner Mongolia section of the Yellow River exhibit diverse land use types, including farmland, grassland, and construction land. To investigate the causes of deformation, InSAR monitoring results are projected onto Sentinel-2 optical imagery. By integrating land use classification, topographic characteristics, and the normalized difference vegetation index (NDVI), a comprehensive analysis is conducted to determine the location and underlying causes of the deformation. If the deformation areas are characterized by sparse vegetation cover and align with natural factors such as riverbank erosion, groundwater level fluctuations, and soil compaction, the deformation is likely to be of natural origin. Conversely, deformation occurring in regions designated as construction land or agricultural fields is more likely to be induced by human activities.

### 3.3. Riverbank boundary extraction using the water index method

Extracting water bodies and subsequently converting them into boundaries is the mainstream approach for delineating water body boundaries, with the water index method being the most effective. This method establishes mathematical relationships between different spectral bands of water bodies to extract water information [30]. It is widely used due to its simplicity and its ability to suppress non-water features in remote sensing images while enhancing water-related information for more accurate extraction. Several commonly used water indices include the normalized difference water index (NDWI), the modified normalized difference water index (MNDWI), and the enhanced water index (EWI) [31–33]. To reduce the impact of image shadows caused by steep riverbanks in certain sections, MNDWI is adopted in this study. It is calculated using the following formula:


MNDWI = (Green−SWIR)/ (Green+SWIR) (5)

where Green represents the green spectral band, and SWIR represents the shortwave infrared band, corresponding to band 3 and band 11 of Sentinel-2 data, respectively.

For water boundary extraction using the water index method, SNAP software is employed for preprocessing remote sensing images, including band composition, radiometric calibration, atmospheric correction, and image cropping. The MNDWI image is generated through band computation, and the histogram bimodal method is applied to determine the threshold for extracting river channel boundaries. However, due to the relatively low resolution of the selected images and the narrow river width in the study area, mixed pixels may affect the accuracy of detailed boundary extraction. To address this issue, a manual visual interpretation approach is adopted to refine the extracted water boundaries. The correction process strictly follows the principle of using a smooth curve to connect the center points of mixed pixels at the interface between water and adjacent land features, ensuring an accurate representation of the river morphology in the study area.

## 4. Results and analysis

### 4.1. Detection of large-scale riverbank deformation areas

**Fig 5:**
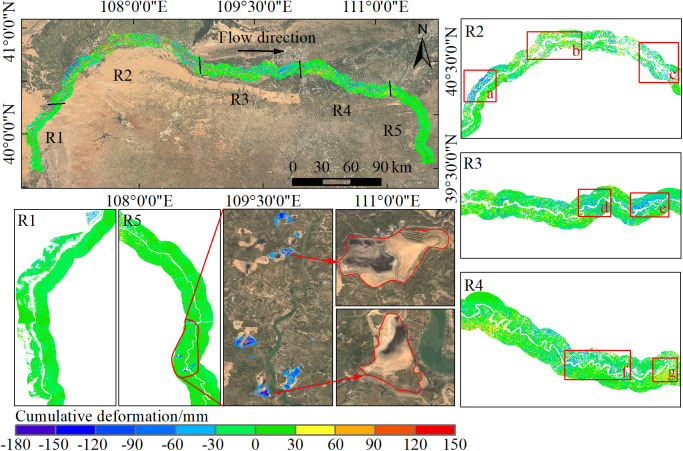
Spatiotemporal distribution of riverbank deformation in the Inner Mongolia section of the Yellow River from 2021 to 2022. The background imagery is based on Sentinel-2 cloudless data (2023) provided by EOX and the Copernicus Data Space Ecosystem (CDSE, https://dataspace.copernicus.eu).

The Inner Mongolia section of the Yellow River was divided into five segments (R1 to R5) based on the distribution of hydrological stations. As shown in Fig 5, the overall riverbank deformation remains stable; however, significant variations exist among different segments. The R1 segment is a straight river section with relatively slow water flow. Severe desertification is observed on both sides of the riverbanks, and the deformation primarily ranges between −30 mm and 30 mm, indicating overall stability. The R2 segment is a braided river section where water flow is dispersed, but localized erosion is intense. Due to agricultural irrigation activities, significant deformation is concentrated in three locations (a, b, and c), covering a wide area with deformation rates exceeding 55 mm/year. Additionally, frequent river channel shifts lead to significant decorrelation, affecting the completeness and accuracy of deformation monitoring. The R3 segment represents a transitional river type, exhibiting characteristics between braided and meandering rivers. Notable deformation is concentrated in curved riverbanks and adjacent farmland areas (d and e), primarily driven by water flow erosion and human activities such as farmland irrigation. The R4 section is a meandering river reach where the riverbank exhibits an overall uplift trend, with deformation magnitudes mainly ranging between 30 and 90 mm. The uplift is likely related to seasonal frost heave and groundwater fluctuations. However, in local areas such as points f and g, subsidence deformation is observed due to factors including river bend scouring and instability of embankment structures. The R5 segment, characterized as a straight river reach with mountainous terrain on both banks, showed only minor overall deformation. However, three zones of substantial subsidence were identified. Sentinel-2 optical imagery revealed that these areas were terraced fields, where heavy rainfall and agricultural activities likely contributed to the pronounced deformation, with the maximum observed subsidence reaching 174 mm.

**Fig 6:**
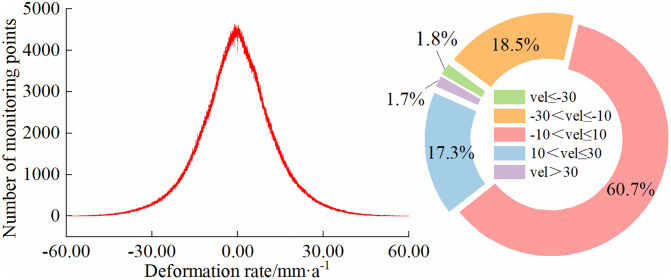
Deformation rate statistics of monitoring points.

As shown in Fig 6, the SBAS-InSAR technique identified a total of 1,677,377 monitoring points for deformation analysis in the Inner Mongolia section of the Yellow River. Among them, 96.5% of the points exhibit deformation rates ranging from −30 mm/a to 30 mm/a, indicating that the riverbanks in this section remain largely stable. However, 3.5% of the monitoring points show significant deformation, primarily concentrated in the R2, R3, and R4 segments. The formation of these significant deformation areas is influenced by multiple factors, including river morphology changes, localized hydraulic erosion, agricultural irrigation activities, and freeze-thaw cycles. These factors interact differently across various river segments, leading to variations in the causes and characteristics of deformation in different regions.

### 4.2. Characteristics of river channel boundary changes

In this study, the 2021 river channel was used as the baseline, and the river centerline was extracted and equally divided. Based on the center points of each segment, 72 cross-sections were established at equal intervals using Thiessen polygons, numbered S1 to S72, with a spacing of 10 km. Sentinel-2 imagery from 2021 to 2023 was selected for analysis, with a spatial resolution of 30 m and cloud coverage below 10%. To accurately observe the mainstream shifting trends, images from April and May, after the freeze-thaw period, were used to extract the main river channel locations. During this period, water levels are at their lowest, effectively minimizing the interference of seasonal and short-term water level fluctuations.

**Fig 7:**
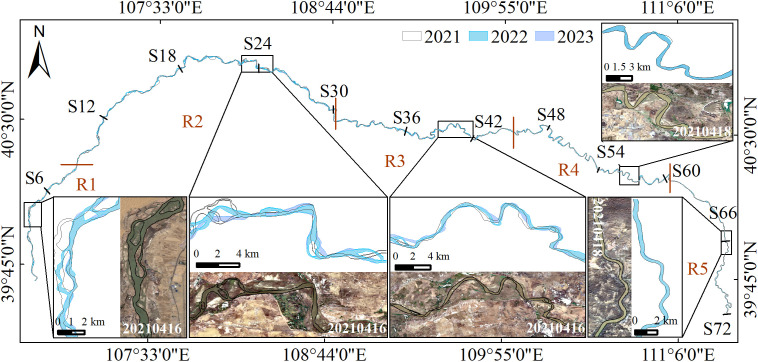
Distribution of river channels and their local characteristics from 2021 to 2023.

Fig 7 demonstrates significant variations in river channel migration characteristics across different reaches of the Inner Mongolia section of the Yellow River.The R1 and R2 segments experience frequent channel migration, with numerous mid-channel bars and significant sediment deposition. Water level fluctuations cause frequent shifts in the riverbank boundaries, significantly increasing the difficulty of deformation monitoring. The R3 segment exhibits well-developed meanders, with an increasing number of river bends, representing a transitional river morphology. The river channel shows a high degree of lateral movement, and lateral erosion is particularly evident. The R4 river segment is located between Baotou City and the Toudaoguai Hydrological Station. In this area, river regulation and bank protection works are relatively well-developed [23], resulting in reduced lateral channel migration and minimal changes in the riverbank. Therefore, this segment is suitable as a primary area for long-term monitoring of riverbank surface deformation characteristics. The R5 segment has shown no significant morphological changes, with minimal river movement, indicating overall stability.

**Fig 8:**
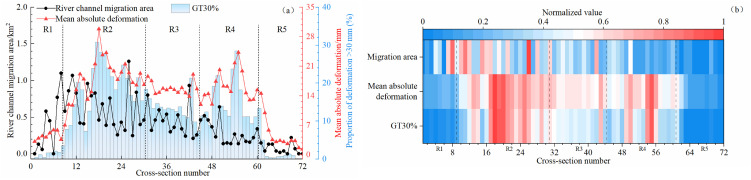
Relationship between channel migration and bank deformation from 2021 to 2023. **(a)** Annual mean variation trends. **(b)** Standardized comparison of three indicators along the cross sections.

To analyze the relationship between channel migration and riverbank deformation, the annual average channel migration area, the annual average absolute riverbank deformation, and the proportion of monitoring points with deformation exceeding 30 mm (GT30%) were calculated for river segments S1-S72 of the Inner Mongolia section of the Yellow River from 2021 to 2023, as shown in Fig 8. During this period, segments R1, R2, and R3 exhibited relatively large migration amplitudes, with migration areas generally exceeding 0.3 km^2^. The R2 segment was the most pronounced, with a maximum migration area exceeding 1.2 km^2^ and a GT30% over 30%. This segment is located in a typical irrigation area within the Hetao Irrigation District, where multiple factors such as irrigation water diversion, groundwater level fluctuations, and flood-season scouring contribute to a relatively loose bank slope structure and evident deformation. Segment R3 also showed strong migration and deformation trends, likely related to channel curvature and localized scouring. Although segment R1 exhibited considerable migration amplitude, its bank slope mainly consists of bedrock with strong resistance to disturbance, resulting in relatively small deformation [23].

Correlation analysis results indicate that the linear relationship between river migration area and riverbank deformation indices is weak (with mean absolute deformation: *R*^2^ = 0.10, *P* < 0.01; with GT30%: *R*^2^ = 0.088, *P* < 0.05), suggesting that river migration amplitude is not the primary controlling factor of riverbank deformation. Polynomial regression results reveal more pronounced quadratic or cubic relationships (*R*^2^ increased to approximately 0.4–0.5) in certain sections, indicating that the influence of river migration on bank deformation exhibits complex nonlinear characteristics rather than a simple linear response. In some reaches, the spatial coincidence between river channel migration boundaries and significant deformation zones detected by InSAR suggests that river migration may still play an important role at the local scale. In contrast, a strong correlation was observed between the mean absolute deformation and GT30% (*R*^2^ = 0.92, *P* < 0.01), implying that as local deformation magnitude increases, the proportion of large-deformation points also rises correspondingly. Therefore, the R4 reach, where the channel migration is relatively stable but the bank deformation is significant, was selected as the primary monitoring section to avoid the interference caused by large-scale channel migration and to better reveal the temporal evolution characteristics of riverbank deformation.

### 4.3. Time-series analysis of typical riverbank deformation

#### 4.3.1. Analysis of riverbank deformation characteristics.

**Fig 9:**
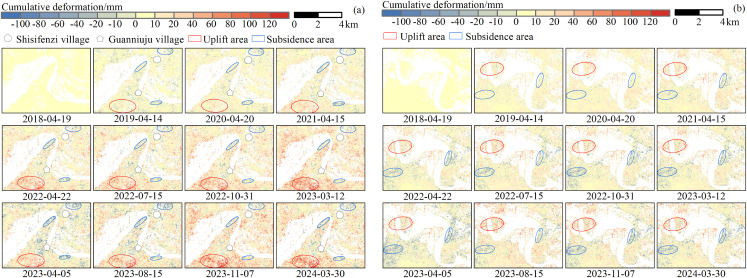
Line of sight (LOS) deformation characteristic map of study area in 2018–2024. **(a)** Shisifenzi area. **(b)** Wenbuhao area.

The Inner Mongolia section of the Yellow River is a typical seasonal permafrost river, where riverbank deformation mainly occurs during the flood season (July to October) and the freeze-thaw period (November to April of the following year), as supported by previous studies and field investigations. Fig 9 presents the riverbank surface deformation in the Shisifenzi and Wenbuhao areas during these two critical periods from 2018 to 2024. In the figure, white areas indicate decorrelation regions, which result from reduced coherence due to factors such as topography, vegetation coverage, and water bodies. The time-series deformation maps reveal that the riverbanks in Shisifenzi exhibit a pattern of overall uplift with localized subsidence. The uplifted areas are mainly concentrated southwest of Guanniuju Village, where deformation has evolved from localized uplift points to a continuous uplift zone of approximately 4.8 km² by 2024, with a maximum cumulative deformation of 125 mm. Meanwhile, subsidence is mainly observed in the bends of the riverbank, particularly at the river meander crest in Shisifenzi and along the embankments near Guanniuju Village. Based on the analysis of meteorological and hydrological data, the subsidence process is likely influenced by a combination of factors, including collapsible settlement caused by heavy rainfall, consolidation due to groundwater level decline, and fluvial erosion along the riverbank. In the Wenbuhao area, surface deformation of the riverbank is dominated by uplift, which is mainly concentrated in the northwestern region and covers a relatively large area, though the significantly uplifted zones are scattered. In 2019, the riverbank exhibited an overall upward trend, while most areas maintained a dynamic balance between uplift and subsidence throughout the year. The northwestern region showed a continuously stable uplift trend, which developed into a pronounced uplift zone by August 2021, with the maximum uplift reaching 87 mm. Subsidence areas were mainly distributed in the central and eastern parts, with a maximum value of 92 mm. Subsidence was particularly pronounced along the outer bends of the river and in sections affected by erosion, where local subsidence exceeded 45 mm.

**Fig 10:**
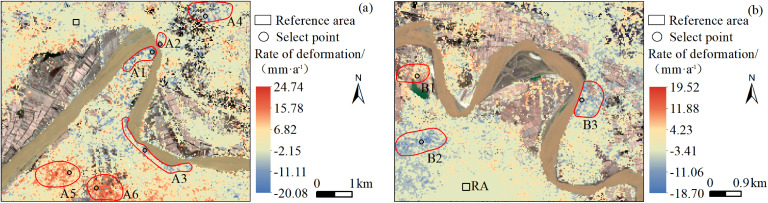
Line of sight (LOS) deformation rate map of study area in 2018–2024. **(a)** Shisifenzi area. **(b)** Wenbuhao area.

Fig 10 presents the deformation rate distribution in the study area from 2018 to 2024. Based on the average LOS deformation rate analysis, areas with an absolute deformation rate of less than 10 mm/a were classified as stable regions, while areas with an absolute deformation rate greater than 10 mm/a were identified as significant deformation regions. According to the clustering characteristics of deformation points, the significant deformation areas in the Shisifenzi region were labeled A1 to A6, while those in the Wenbuhao region were labeled B1 to B3. In the Shisifenzi region, the annual average deformation rate ranged from −20.1 mm/a to 24.8 mm/a. The A1 and A2 areas, located at the meander crest of Shisifenzi, experience rapid water flow and a constricted river channel, leading to significant bank subsidence, with an average subsidence rate of 15.1 mm/a and 10.2 mm/a, respectively. The A3 area, positioned on the right riverbank near Guanniuju Village, exhibits a subsidence zone extending approximately 2.6 km, where frequent water scouring has caused notable bank settlement, with an average subsidence rate of 11.7 mm/a. The A4 area, near Shisifenzi Village, recorded the highest subsidence rate of 15.2 mm/a. In contrast, the A5 and A6 areas display large-scale uplift, with deformation rates ranging from 12.1 mm/a to 24.8 mm/a, forming a spatially continuous uplift zone. Unlike the other areas, A4, A5, and A6 are far from the riverbank and located in agricultural zones, where hydraulic influences are minimal. The significant deformation in these areas is likely closely related to groundwater level fluctuations and agricultural activities. In the Wenbuhao region, the annual average deformation rate ranged from −18.7 mm/a to 19.5 mm/a. The B1 area, located at the riverbank boundary, exhibits notable uplift, with an average uplift rate of 16.7 mm/a. The B2 area experiences large-scale subsidence, with a maximum subsidence rate of 14.9 mm/a. The B3 area, situated within a birch forest on the concave bank of the river, demonstrates a gradual annual subsidence trend. Although vegetation root systems enhance riverbank stability [34], the combined effects of water erosion and freeze-thaw cycles have resulted in progressive surface subsidence, with a subsidence rate of approximately −13.5 mm/a. Overall, the subsidence and uplift patterns shown in Fig 10 exhibit clear spatial heterogeneity. Areas near the outer edges of river bends (A1, A2, A3, and B3) are significantly influenced by fluvial erosion. Hydrodynamic disturbances in these zones often lead to reduced slope stability and shallow soil compaction-induced subsidence, which may not be fully mitigated by vegetation cover or engineered embankments. In contrast, the notable uplift areas located farther from the river channel (A4, A5, A6, and B2) are mostly situated within agricultural zones. Deformation in these areas may be associated with seasonal fluctuations in groundwater levels, frost heave effects induced by irrigation, or farmland backfilling activities.

#### 4.3.2. Time-series deformation characteristics of feature points.

**Fig 11:**
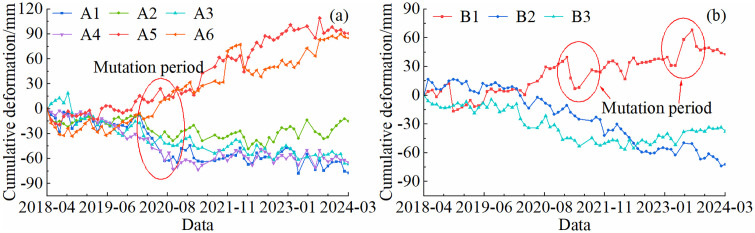
Cumulative deformation curve of feature points. **(a)** Shisifenzi area. **(b)** Wenbuhao area.

In areas exhibiting significant deformation, representative points with deformation rates close to the regional average and high temporal coherence were selected for time-series deformation analysis. The cumulative deformation of these feature points in the Shisifenzi and Wenbuhao regions is shown in Fig 11. The results indicate that riverbank surface deformation exhibits an alternating pattern of uplift and subsidence, characterized by seasonal variability and abrupt changes. From December to March each year, the deformation areas generally show an uplift trend, whereas during the melting period from March to April and the heavy rainfall months from June to August, the subsidence rate increases significantly. Based on the deformation patterns, the deformation process can be categorized into relatively stable periods and abrupt change periods. Stable periods are characterized by continuous and steady deformation rates or periodic variations, whereas abrupt change periods are marked by a sudden acceleration in deformation rate and a significant increase in deformation magnitude.

As shown in Fig 11, the maximum cumulative deformation at feature points in the A1-A6 areas was −77.8 mm, −48.6 mm, −61.3 mm, −72.6 mm, 109.3 mm, and 90 mm, respectively. Among them, A1–A4 are subsidence zones, where rapid subsidence occurred from March 2020 to October 2020, with a total subsidence exceeding 30 mm in each location. Based on meteorological data analysis, precipitation from July to September 2020 was significantly above average, and the accelerated subsidence during this period coincided temporally with extreme rainfall events. Considering that no large-scale geological activity or engineering disturbances occurred in the area during this anomalous period, the subsidence is inferred to have been primarily driven by thaw-induced soil settlement resulting from spring ice-water phase changes, as well as increased pore water pressure and collapsible soil compression caused by intense autumn rainfall. In contrast, the A5 and A6 regions did not exhibit significant subsidence during the same period, maintaining a relatively stable uplift trend. During the relatively stable period, the A2 area initially showed a stable deformation trend, but gradual uplift was observed starting in October 2022. The remaining three subsidence areas displayed similar trends, characterized by gradual and continuous subsidence. For the B1-B3 areas, the maximum cumulative deformation reached −68.1 mm, −73.7 mm, and −56.4 mm, respectively. The B1 region is dominated by uplift, with cumulative deformation showing a gradually increasing trend. Two pronounced fluctuations, each with an amplitude of approximately 23 mm, occurred around April 2021 and June 2023. Both events coincided with periods of rapid springtime river water level rise and freeze-thaw transitions, and are therefore inferred to be closely related to abrupt increases in river water levels and rapid changes in soil moisture conditions. The B2 area exhibited relatively stable deformation, following a consistent subsidence trend. In the B3 area, deformation was initially minor, but between February 2020 and May 2021, subsidence was most pronounced, with a total settlement of 44 mm. After 2023, the subsidence rate gradually decreased.

### 4.4. Accuracy assessment and validation

**Fig 12:**
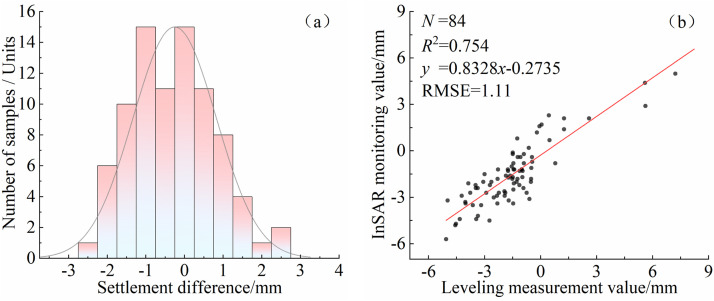
Distribution of cumulative settlement differences and linear relationship. **(a)** Difference distribution. **(b)** Linear relationship.

To verify the reliability of InSAR technology for riverbank deformation monitoring, three settlement pressure sensors were installed near the riverbank boundary in both the Shisifenzi and Wenbuhao areas, with an approximate spacing of 50 meters between sensors. These sensors were used to more accurately capture localized surface subsidence characteristics. The selection of leveling benchmarks was based on the InSAR monitoring results from 2021−2022 and field investigations. Priority was given to relatively stable riverbank sections located in meandering areas less affected by water flow erosion, in order to ensure the representativeness and safety of the monitoring data. The coordinates of the central leveling point in the Shisifenzi area are 111°2′46.2″E, 40°17′39.9″N, and those in the Wenbuhao area are 110°46′14.9″E, 40°17′30.5″N, as shown in Fig 1. Since only ascending orbit data from Sentinel-1 were available in this study, it was not possible to derive the full 3D displacement components through orbit combination. Therefore, it was assumed that the surface deformation in the study area occurred primarily in the vertical direction. Based on this assumption, the LOS (line-of-sight) displacements were projected onto the vertical direction for comparison with leveling measurements. The leveling measurements were conducted from October 2023 to March 2024, during which radar images from the same period were selected, resulting in a total of 84 monitoring data points. As shown in Fig 12, the mean and standard deviation of the differences between the cumulative settlement values obtained from InSAR monitoring and leveling measurements were 0.84 mm and 1.16 mm, respectively. The distribution of these differences approximately follows a normal distribution. A linear regression analysis was performed between the leveling measurements and InSAR monitoring results, revealing a significant linear correlation, with a coefficient of determination (*R*^2^) of 0.754 and a root mean square error (RMSE) of 1.11 mm. The strong agreement between the two methods confirms the reliability and consistency of InSAR monitoring for riverbank deformation.

To further quantify the uncertainty in surface deformation rate estimates, this study takes the Shisifenzi area as example and conducts a statistical analysis of the root mean square error (RMSE) of time-series residuals at monitoring points within the study area. RMSE reflects the degree of dispersion of residuals in the time-series deformation data. A smaller RMSE value indicates a higher consistency between the deformation time series and the model fitting, implying more reliable inversion results.

**Fig 13:**
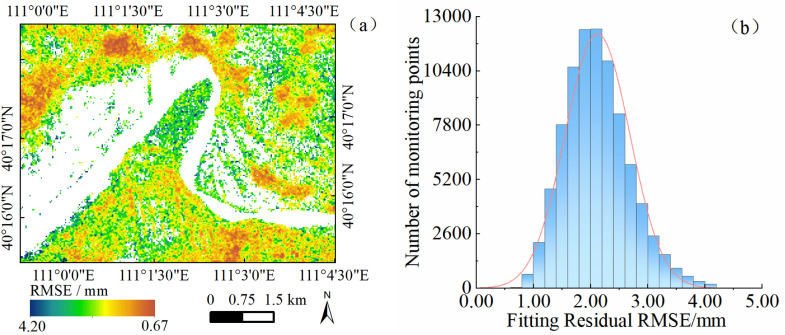
Spatial distribution and statistical histogram of RMSE in the study area. **(a)** Spatial distribution map. **(b)** Statistical histogram.

As shown in Fig 13, the RMSE values across the study area range from 0.67 to 4.20 mm, with a generally uniform spatial distribution. In areas with stable surface conditions, such as river embankments and villages, the RMSE values are generally low, indicating high signal-to-noise ratios in the interferograms and stable time-series inversion results. In contrast, higher RMSE values are observed in regions with significant vegetation variation or proximity to water bodies, where radar signals are more susceptible to interference. This is consistent with the commonly observed reduction in InSAR accuracy under complex surface conditions. The RMSE histogram indicates an approximately normal distribution, with more than 95% of the monitoring points exhibiting RMSE values below 1.28 mm and a regional average of 2.12 mm. These results demonstrate the high internal consistency of the deformation time series obtained in this study. In addition, external accuracy validation using leveling measurements further confirms the reliability and methodological adaptability of the SBAS-InSAR technique for monitoring surface deformation in riverbank areas.

## 5. Discussion

### 5.1. Analysis of the impact of vegetation cover on InSAR coherence

**Fig 14:**
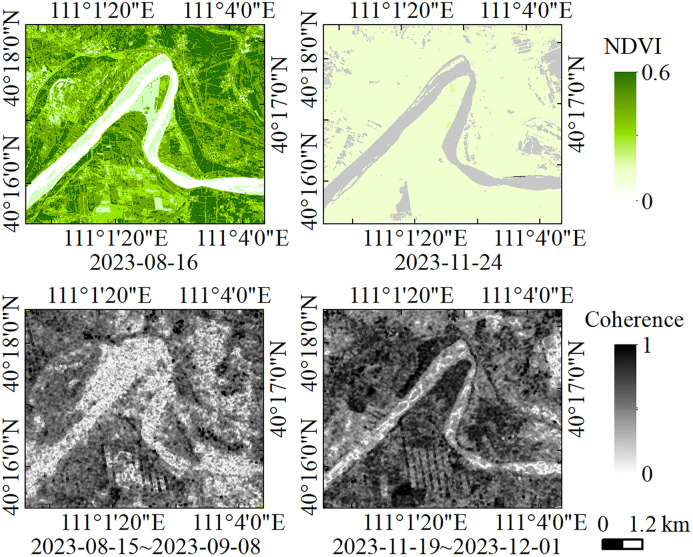
Comparison of NDVI and InSAR coherence.

Vegetation cover is a critical factor affecting InSAR coherence, particularly in farmland areas and densely vegetated riverbanks. To assess the extent to which vegetation influences deformation monitoring, images from periods of high vegetation cover (flood season) and low vegetation cover (winter) were selected. The Normalized Difference Vegetation Index (NDVI) was computed and compared with InSAR coherence maps from the same periods, as shown in Fig 14. On August 16, 2023, the NDVI values of the riverbanks in the study area were generally above 0.4, with some areas reaching 0.6. However, InSAR coherence remained above 0.5 in most regions, with only a slight decrease in certain farmland areas with high NDVI values. By November 24, 2023, as vegetation withered, NDVI values dropped below 0.3, and coherence significantly increased. This comparative validation indicates that, although vegetation cover can locally interfere with coherence, its overall impact is limited. In particular, in relatively flat riverbank areas with uniform vegetation types, the effect of vegetation on deformation monitoring is minimal.

### 5.2. Analysis of deformation causes

#### 5.2.1. Relationship between riverbank deformation and precipitation.

**Fig 15:**
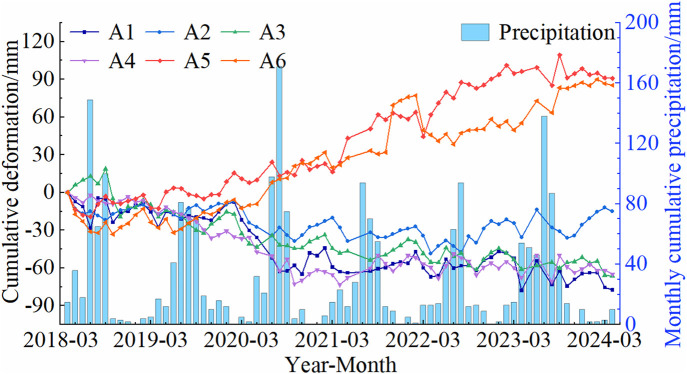
Relationship between settlement feature points and precipitation.

Precipitation is an important factor influencing riverbank surface deformation. Intense rainfall can significantly alter soil moisture conditions, reduce its cohesion and shear strength, and may lead to increased pore water pressure, resulting in soil consolidation or collapsible settlement [35]. At the same time, surface runoff can accelerate the erosion of topsoil in localized areas, weakening slope stability and thereby increasing the risk of local collapse or subsidence [36]. As shown in Fig 15, the maximum monthly cumulative precipitation in the Shisifenzi area reaches approximately 155 mm, while in most months, precipitation remains below 30 mm. Analysis of the cumulative precipitation data from 2018 to 2024 and the cumulative settlement of representative points indicates that when monthly precipitation is below 50 mm, rainfall mainly affects the moisture content of surface soils, with limited particle swelling, and has little impact on the overall deformation trend. During the flood season from July to September, precipitation increases significantly, and in certain years (e.g., 2018, 2020, and 2023), extreme rainfall events correspond closely with abrupt increases in settlement rates in the A1-A3 regions. This suggests that short-term heavy rainfall may trigger rapid soil consolidation and collapsible settlement, leading to the formation of sudden deformation periods. In the A5 and A6 regions, which show a sustained uplift trend, settlement also occurs during heavy rainfall, likely related to foundation consolidation, collapsible soil behavior, and changes in pore water pressure induced by rainfall infiltration. Since the study area is predominantly characterized by gentle slopes and plains, rainfall-induced surface deformation mainly manifests as vertical settlement. Although some slope areas may present a risk of lateral movement, most riverbanks have been engineered for protection, so the primary deformation remains vertical settlement, with lateral displacement having a relatively minor effect.

#### 5.2.2. Relationship between riverbank deformation and water levels.

**Fig 16:**
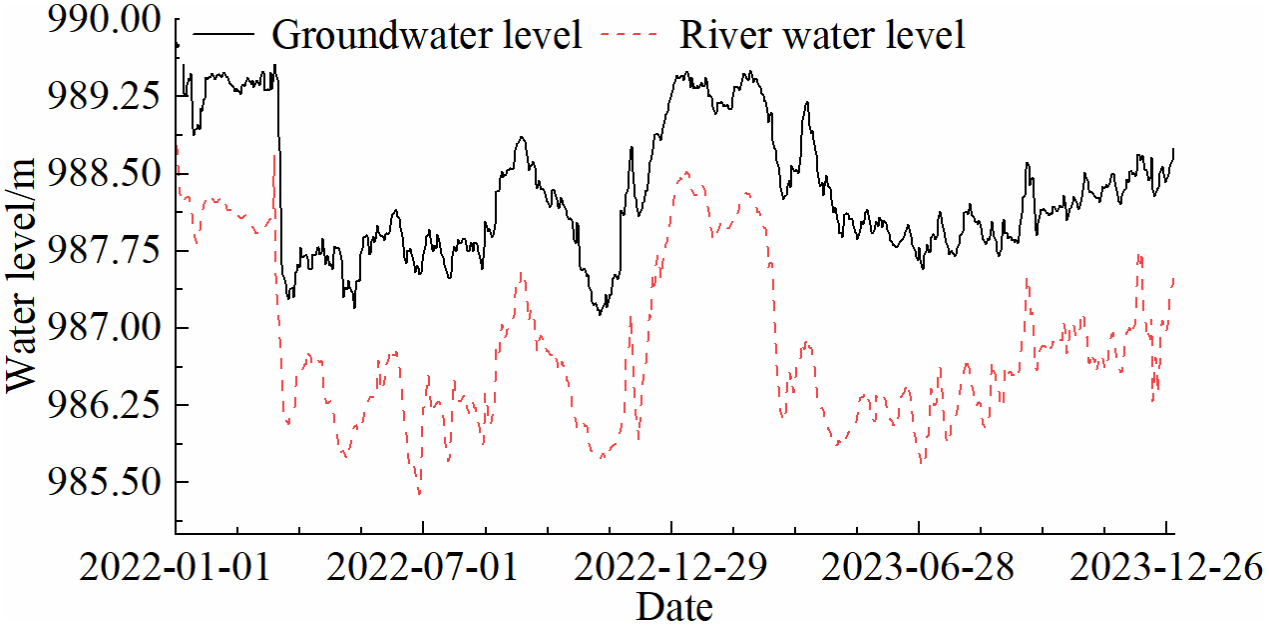
Comparison of river water level and groundwater level changes from 2022 to 2024.

In the Shisifenzi area, strong solar radiation leads to high surface water evaporation, and natural precipitation during the agricultural season is limited. Consequently, local farmland production heavily depends on irrigation from the Yellow River and groundwater recharge, making the coupling between groundwater levels and river stage a critical factor influencing riverbank deformation. Groundwater level data were obtained from piezometers installed at the meander crest in Shisifenzi, while river water level data were collected from the Toudaoguai hydrological station, located 5 km downstream of the meander crest. As shown in Fig 16, due to topographic differences, the groundwater level at the meander crest is approximately 1.41 m higher than the river water level recorded at the hydrological station. However, the overall variation trends of both water levels remain highly consistent. Each year, both groundwater and river water levels peak at approximately 989.3 m in early January, followed by a rapid decline to 988.2 m by mid-March, after which they stabilize for an extended period. The fluctuations in groundwater levels are primarily influenced by river water level changes. When river water levels are low, groundwater from the riverbank slope flows into the river as discharge. Conversely, when river water levels rise, the river provides lateral moisture recharge to the riverbank slope. Therefore, their temporal fluctuation patterns remain highly synchronized.

Building on the water level relationship established in Fig 16,17 further analyzes the relationship between riverbank deformation and groundwater levels from April 2018 to April 2024. Groundwater levels at the river bend crest were estimated over a long-term period using river stage data from the Toudaoguai Hydrological Station, with A1 (convex bank near the water side), A2 (concave bank scouring section), and A4 (farmland approximately 1.2 km from the river) selected as representative monitoring points. In the Shisifenzi area, groundwater exhibits distinct seasonal patterns. Each winter (November to April), groundwater levels rise to an average of 990.7 m. The combination of high water levels and soil freezing increases soil moisture, resulting in seasonal frost heave and a gradual uplift trend. During the spring thaw and water level recession, soil structure softens and moisture content decreases, causing local subsidence. This phenomenon is particularly pronounced in the A1 and A2 areas near the river, where the deformation magnitude is relatively large. In contrast, the A4 area, located farther from the river, shows smaller overall deformation; however, significant changes are still observed during some high-water periods, which may be associated with agricultural irrigation or long-term fluctuations in groundwater levels.

**Fig 17:**
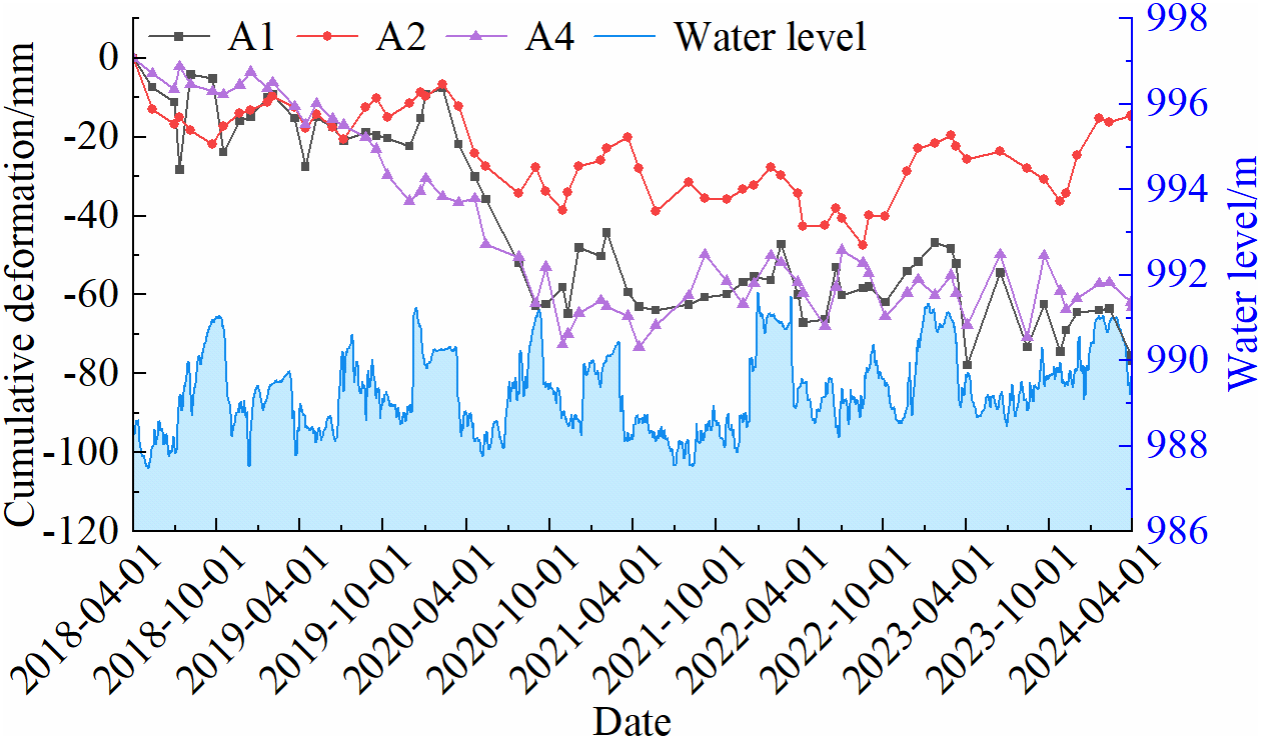
Relationship between cumulative deformation of characteristic points and water level changes.

### 5.3. Applicability analysis of the research method

In recent years, SBAS-InSAR technology has achieved significant advancements in various fields, including landslide monitoring, urban subsidence, thaw settlement in permafrost regions (e.g., the Tibetan Plateau), and hydraulic engineering projects (e.g., reservoirs and breakwaters), owing to its large-scale coverage and high precision. However, research on seasonally frozen soil riverbank deformation remains relatively limited. Unlike permafrost regions, where deformation is primarily controlled by thaw settlement [37,38], riverbanks in seasonally frozen regions experience annual freeze-thaw cycles and are influenced by vegetation cover changes, fluvial erosion, and groundwater level fluctuations, making the deformation mechanisms more complex and the monitoring process more challenging. This study applies SBAS-InSAR technology to monitor riverbank deformation along the Inner Mongolia section of the Yellow River. The Sentinel-1A data were validated against leveling measurements, confirming the reliability of SBAS-InSAR in complex environments and providing a new technical perspective for riverbank disaster prevention and mitigation.

Although the study results show good agreement with leveling measurements, demonstrating the reliability of SBAS-InSAR for riverbank deformation monitoring, certain limitations must still be acknowledged. First, due to the availability of open Sentinel-1A data, only ascending-track images could be obtained in the study area, making it difficult to perform ascending-descending track joint inversion. This limits the recovery and directional interpretation of the full 3D deformation field. Although the study area is generally flat, and single-geometry observations can adequately reflect the primary vertical deformation, constraints on potential horizontal displacement are lacking, restricting a comprehensive reconstruction and interpretation of the 3D deformation field. Second, integration of multi-source data remains limited. This study primarily relies on medium-resolution C-band Sentinel-1 SAR images (approximately 10–20 m) for large-scale time-series monitoring, combined with precipitation and river stage data to explore the relationship between external environmental factors and riverbank deformation. These auxiliary data are currently used mainly for qualitative analysis; systematic quantitative modeling and validation of the spatiotemporal relationships between precipitation and subsidence rates, as well as the lag effects of water level fluctuations on bank deformation, are still lacking. This limitation restricts deeper insights into the driving mechanisms of deformation. Future studies could incorporate high-spatial-resolution radar data (e.g., TerraSAR-X with a resolution of 1–3 m) and ascending-descending track imagery to improve monitoring precision and enhance the identification of deformation characteristics.

In addition, the surrounding area of the study region is a typical agricultural zone, where activities such as irrigation from the Yellow River, channel excavation, and other sudden human activities may trigger local nonlinear or abrupt surface deformations. Currently, the primary method for identifying such activities relies on visual interpretation of optical imagery, which has limited quantification ability and makes it difficult to precisely quantify the impact of human disturbances. Future research could utilize Sentinel-2 optical imagery to extract NDVI indices and land use classification maps, preliminarily delineating the boundaries of agricultural activities. Furthermore, by combining SAR intensity and phase information with machine learning and other intelligent recognition methods, automated extraction of features such as irrigation canal networks and agricultural construction areas could be achieved, providing more detailed data support for the analysis of riverbank deformation mechanisms.

From a practical perspective, the significant deformation river segments identified in this study (e.g., R2, R3, and R4) can serve as priority areas for riverbank reinforcement and hydraulic engineering protection. The results provide data support for flood prevention and disaster mitigation, riverbank ecological restoration, and agricultural irrigation planning, while also offering a scientific basis for local authorities to formulate riverbank management and risk control policies. In the future, a riverbank deformation early-warning system could be established based on InSAR time-series monitoring, enabling the translation of monitoring research into engineering and policy applications.

## 6. Conclusions

This study systematically monitored riverbank surface deformation along the Inner Mongolia section of the Yellow River using SBAS-InSAR, identified the major deformation areas and their spatiotemporal distribution, and verified the applicability and reliability of this technique under complex permafrost-region terrain conditions. The results indicate that InSAR-based monitoring is highly reliable in permafrost regions with complex geomorphology and can effectively reveal the spatiotemporal heterogeneity of riverbank surface deformation. Although the overall riverbanks in the study area are relatively stable, significant differences exist among different river segments. River channel migration exhibits a spatially positive correlation with slope deformation, with variations mainly influenced by riverbank soil composition and its physical-mechanical properties. Under the combined effects of freeze-thaw cycles and flood-season water flow, uplift primarily occurs in cultivated areas distant from the river, whereas subsidence is concentrated in regions subject to strong flow erosion. In addition, riverbank deformation is mainly controlled by groundwater level fluctuations; while minor precipitation has limited influence, extreme rainfall events may exacerbate subsidence risks. In summary, SBAS-InSAR demonstrates high reliability for monitoring riverbank deformation in permafrost regions, providing theoretical support and technical guidance for risk assessment, disaster early warning, and mitigation planning for riverside levees. In particular, under the context of frequent extreme climate events, this study contributes to the identification of potential high-risk sections and enhances flood prevention and disaster mitigation capabilities along the riverbanks.
